# The Pathology of Paralysis with Muscular Degeneration (Paralysie Myosclérotique)

**Published:** 1868-10

**Authors:** 

**Affiliations:** Boulogne


### THE PATHOLOGY OF PARALYSIS WITH MUSCULAR DEGENERATION (PARALYSIE MYOSCLÉROTIQUE).

By Dr. DUCHENNE, of Boulogne. Communicated to the Pathological Society of London by Mr. LOCKHART CLARKE, F.R.S.

The clinical facts which serve as a basis for studying the symptomatology of the disease show that paralysis with degeneration (*sclerosie*) of muscles, or with apparent hypertrophy of muscles, is marked, in general, by three distinct periods or stages—a stage of paralysis, a stage of hypertrophy, and a stage in which the paralysis becomes general.

1. The first stage is manifested either at the time when the children should begin to walk, or some years after they have begun. In the former case, although the conformation of these children be quite normal, and although, while lying down or in the arms of their mothers or their nurses, they appear to possess their natural share of mobility, yet, when they arrive at the age of twelve or fourteen months, if an attempt be made to stand them on their legs, they immediately fall down. It is not until they have attained the age of two or three years that they are able to stand upright or to walk, and even then they require support. In the latter case, after these children have walked well for several years, it is remarked that, either spontaneously or subsequently to some convulsions, they are soon fatigued by standing or walking; that, without some support, they find these operations become more and more difficult and painful, and that they are subject to frequent falls. Whatever may have been the age at which the malady first made its appearance, it is soon observed that, in order to maintain their equilibrium while standing or walking, all these children bend themselves very much backward and keep their legs very much apart; that at each step they incline laterally towards the leg which rests on the ground—a movement which produces a characteristic balancing of the body during progression.

2. The second stage is announced, in general, some months, and even two years, after the beginning of the muscular weakness, by a progressive swelling or enlargement of the gastrocnemii, then of the glutei and the lumbar muscles of the spine. This apparent hypertrophy occurs sometimes in nearly all the muscles that have been affected by paralysis; but, in general, it does not, and it may even be limited to a very small number of the muscles. The extension, to a greater or less extent, of the apparent hypertrophy of the muscles may constitute different varieties of this kind of paralysis. The hypertrophied muscles are firm and elastic; they become very hard while they contract, and show all the relief or projection which properly belongs to their contracted state; they then appear to form a hernial protrusion through the integument, which is very thin. Moreover, their great size shows off the apparent smallness and delicacy of the joints at the knee, ankle, etc.

In one case that came under my observation, both the weakness and the muscular hypertrophy appear to have shown themselves simultaneously. According to the information given by the mother, the child was very large at its birth. But this information is insufficient; we ought to know whether the great size of the body and of the limbs was or was not due rather to the abundance of subcutaneous adipose tissue than to the volume of the muscular masses.

The increase in the size of the muscles does not appear to add to their weakness. This is so far from being the case, that the muscles of the calves, which are always the most hypertrophied, are those which are found to have relatively the greatest power.

The morbid phenomena above described may remain in the same state for years—sometimes until a tolerably advanced period of youth.

A new stage of the disease, and the last one, is manifested by a gradual increase in the severity, and a more general extension, of the paralysis. The young patients can no longer stand upright; they always remain in the recumbent posture, without any power to change the position in which they may be placed; and the upper extremities, if they have not hitherto been affected, soon lose all their movements. With this aggravation of the paralysis, hypertrophied muscles may sometimes be seen to melt, as it were, away, and then all the limbs and the trunk become atrophied *en masse.* Although in this stage the patients are reduced to a state of great weakness, they may nevertheless live for a tolerably long time. They are cut off by some intercurrent disease.

Mr. Clarke adds:

Dr. Duchenne informs me that he has been studying this disease for the last eleven years, and is now preparing a small work on the subject. Several cases have been recorded on the continent, and *post mortem* examinations have been made. Eulenburg and Cohnheim examined the body of a child which died of this disease at the age of thirteen. They found the electro-muscular contractility everywhere intact. Nothing abnormal was discovered in the nervous and vascular systems. To the touch, the muscles of the lower limbs gave the sensation of a doughy and inelastic mass. They were marked with stripes or striæ of a yellow or yellowish-white appearance. On section, they shone with a kind of greasy light. At certain points they could not be distinguished by the naked eye from the subcutaneous adipose tissue. The muscles of the upper extremities presented a similar kind of structure; but they were much atrophied, as were also those of the trunk. Under the microscope, those especially of the lower extremities seemed to be filled with adipose tissue; but the muscular tissue itself was not altered. Griesinger and Billroth had already observed a similar state in the living subject. It is rare, however, to find any oily particles in them. (*Verhandl. d. Berliner Med. Ges.*, *1*, *101 205.*) Heller, who examined two brothers that died of this disease, seems to consider it as a kind of fatty degeneration, for he calls it lipomatus. (*Deutsches Archiv-f. Klin. Med.*, *1*, *616-627.*) Seidel, also, records three cases belonging to the same family, under the term of "lipomatous atrophy of the muscles, or muscular atrophy."—*British Medical Journal*, Dec. 14, 1867.

(Since the date of this communication, the article promised by Duchenne has been published in the *Archives generales de Medicine*, Jan.–May, 1868.)

